# Improvement of an automated protein crystal exchange system PAM for high-throughput data collection

**DOI:** 10.1107/S0909049513021067

**Published:** 2013-10-01

**Authors:** Masahiko Hiraki, Yusuke Yamada, Leonard M. G. Chavas, Soichi Wakatsuki, Naohiro Matsugaki

**Affiliations:** a Structural Biology Research Center, Photon Factory, Institute of Materials Structural Science, High Energy Accelerator Research Organization, 1-1 Oho, Tsukuba, Ibaraki 305-0801, Japan; b Department of Photon Science, SLAC National Accelerator Laboratory, 2575 Sand Hill Road, MS 69, Menlo Park, CA 94025-7015, USA; c Department of Structural Biology, School of Medicine, Stanford University, Beckman Center B105, Stanford, CA 94305-5126, USA

**Keywords:** protein crystallography, sample-exchange robot, automated system

## Abstract

Photon Factory Automated Mounting system (PAM) protein crystal exchange systems are available at the following Photon Factory macromolecular beamlines: BL-1A, BL-5A, BL-17A, AR-NW12A and AR-NE3A. The beamline AR-NE3A has been constructed for high-throughput macromolecular crystallography and is dedicated to structure-based drug design. The PAM liquid-nitrogen Dewar can store a maximum of three SSRL cassettes. Therefore, users have to interrupt their experiments and replace the cassettes when using four or more of them during their beam time. As a result of investigation, four or more cassettes were used in AR-NE3A alone. For continuous automated data collection, the size of the liquid-nitrogen Dewar for the AR-NE3A PAM was increased, doubling the capacity. In order to check the calibration with the new Dewar and the cassette stand, calibration experiments were repeatedly performed. Compared with the current system, the parameters of the novel system are shown to be stable.

## Abstract 2

A special liquid-nitrogen Dewar with double capacity for the sample-exchange robot has been created at AR-NE3A at the Photon Factory, allowing continuous fully automated data collection. In this work, this new system is described and the stability of its calibration is discussed.

## 1. Introduction

Automated sample-exchange robots are indispensable for efficient usage of macromolecular crystallography beamlines. Once users set samples on the automated system inside an experimental hutch before their experiments, the samples can be handled by the automated system during beam time. For example, the users select a sample from the uploaded list and click a button on the GUI of the beamline control system. The robot then exchanges samples inside the experimental hutch. If the users securely login to the beamline control system from outside of the synchrotron facility, they do not need to visit the facility for their experiments. The users send their samples to the facility by mail beforehand, the support staff at the beamlines insert samples into the robot before the beam time, and then the users can carry out their experiments by remote control. In addition, the sample-exchange robot and automated centring software make fully automated data collection possible.

In order to handle a large number of samples efficiently, various sample-exchange systems have been developed and are currently in use at many synchrotron facilities: Stanford Auto-Mounting (SAM) system developed by SSRL (Cohen *et al.*, 2002 ▶), the system by DORIS (Karain *et al.*, 2002 ▶), the system by ALS (Snell *et al.*, 2004 ▶), Cryogenic Automated Transfer System (CATS) by ESRF (Ohana *et al.*, 2004 ▶; Jacquamet *et al.*, 2009 ▶), SPring-8 Precise Automatic Cryo-sample Exchanger (SPACE) by SPring-8 (Ueno *et al.*, 2004 ▶) and Sample Changer (SC3) by EMBL-Grenoble (Cipriani *et al.*, 2006 ▶). Several robots are commercially available, such as Automated Crystal Transport Orientation and Retrieval robot (ACTOR) from Rigaku, Marresearch Cryogenic Sample Changer (marcsc) from Marresearch GmbH, BRUNO from Bruker, CATS from IRELEC and G-Rob from NatX-ray.

At the Photon Factory (PF), the sample-exchange systems have been in operation at BL-5A and AR-NW12A (Chavas *et al.*, 2012 ▶) since 2006. These systems were developed based on the SAM and modified in order to fit our beamlines (Hiraki *et al.*, 2007 ▶). In addition, we developed double tongs (Hiraki *et al.*, 2008 ▶) to reduce the time required for sample exchange. We named the sample-exchange robot with double tongs ‘PAM’ (PF Automated Mounting system) and installed PAM at BL-17A (Igarashi *et al.*, 2007 ▶). PAM was installed at AR-NE3A (Yamada *et al.*, 2010 ▶) in collaboration with Astellas Pharma Inc., a pharmaceutical company. Subsequently, another PAM was installed on a newly built microfocus lower-energy beamline, BL-1A. The double tongs were already implemented at BL-5A and AR-NW12A, raising the number of available PAMs at the PF macromolecular crystallography beamlines to five. At AR-NE3A especially, large-scale fully automated experiments are conducted frequently and an increase in capacity is expected.

Here we describe a major improvement to the PAM, that is, the development of a larger liquid-nitrogen Dewar. Furthermore, we present the characteristics of the system with the developed Dewar based on experimental results.

## 2. Automated protein crystal exchange system PAM

### 2.1. System overview

**Figure 1:**
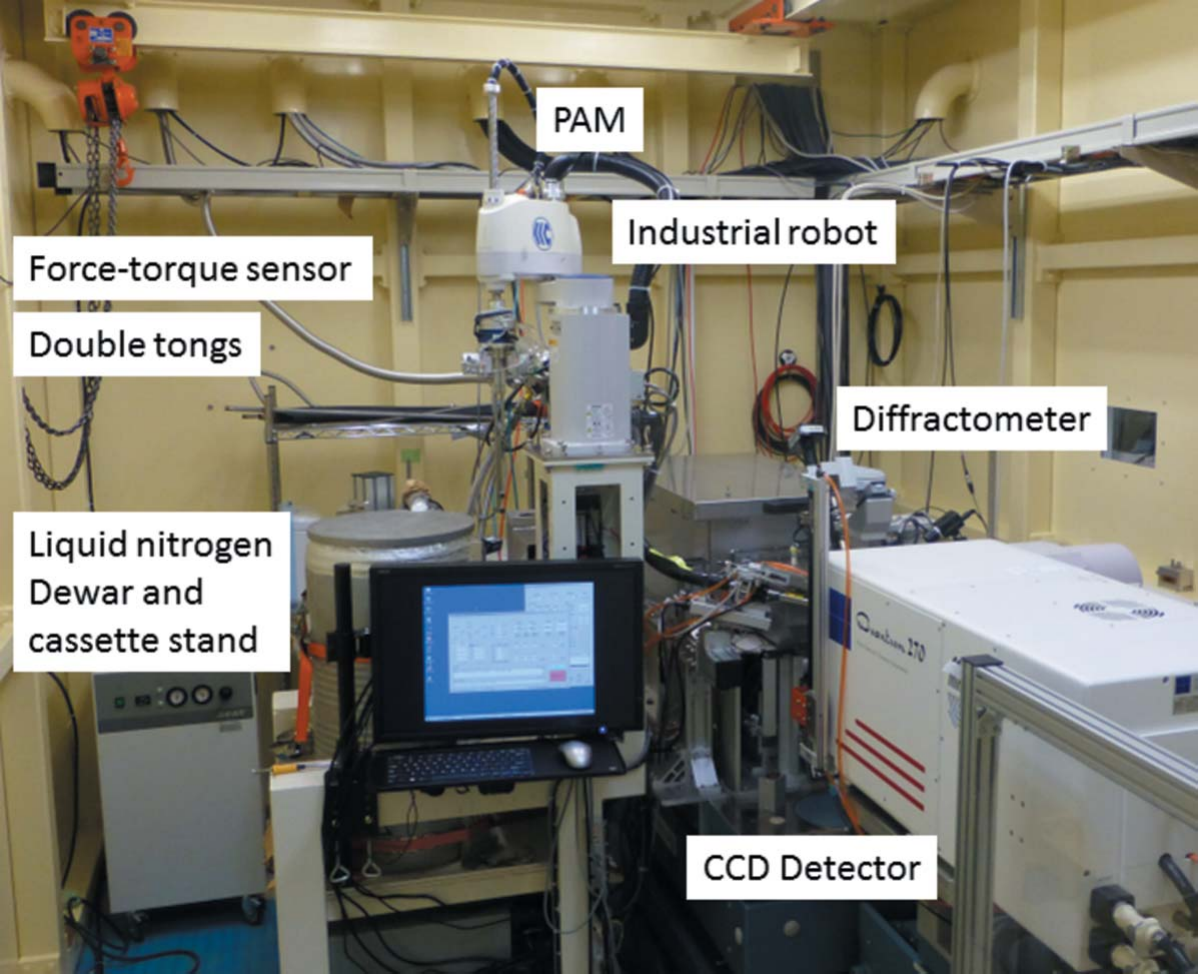
Inside of the experimental hutch of the AR-NE3A beamline and the automated protein crystal exchange system PAM.

**Figure 2:**
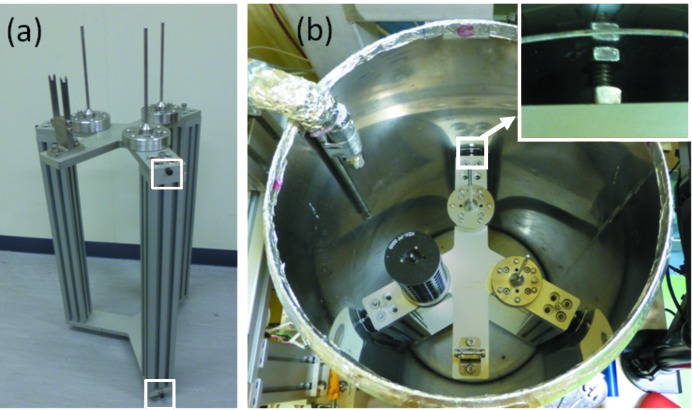
(*a*) Cassette stand. Screws in white boxes are used for fixing the cassette stand in the Dewar. (*b*) Inside of the PAM liquid-nitrogen Dewar.

For efficient use of beam time *via* fully automated experiments and remote control, we have installed the protein crystal exchange system PAM at all the macromolecular crystallography beamlines at the PF. PAM consists of a four-axis industrial robot and its controller (same series as the SAM), double tongs, a force-torque sensor and a commercial liquid-nitrogen Dewar 3K from Taylor Wharton, as shown in Fig. 1 ▶. The industrial robot and the Dewar are fixed tightly to a frame. The force-torque sensor is connected between the *z*-axis of the robot and the double tongs, and is used for detecting collisions and calibrating the cassette stand within the Dewar (Fig. 2 ▶). The cassette stand (Fig. 2*a* ▶) is inserted into the Dewar, and it is fixed weakly by pushing the Dewar wall by the screw heads (Fig. 2*b* ▶) attached on the side of the top and the bottom plates of the cassette stand [shown by the white boxes in Fig. 2(*a*) ▶].

The protein crystals captured by cryoloops are stored in the SSRL cassette or the Universal V1-puck (Uni-puck). The SSRL cassettes and the Uni-puck can hold 96 and 16 cryoloops, respectively. There are three cassette locations within the liquid-nitrogen Dewar of the PAM for the SSRL cassette or an adapter cassette containing four Uni-pucks. Therefore, the capacity of the cryoloops is 288 samples.

### 2.2. Operation status

**Figure 3:**
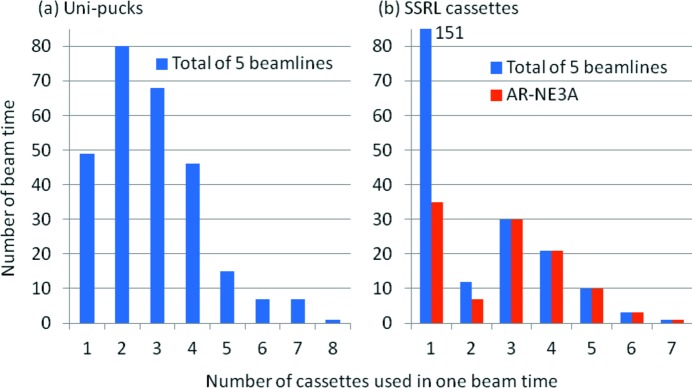
Distribution of the number of cassettes carried in one beam time. The number of cassettes is estimated from the number of mounted samples. For example, three SSRL cassettes correspond to 193–288 samples.

Sample exchange by the robots was started in 2006 at BL-5A and AR-NW12A. Approximately 60000 samples have been mounted by the robots as of 31 December 2012. Firstly, the samples were inserted using the SSRL cassettes. In addition, we modified the PAM control software and beamline control software for the Uni-puck; users have been able to carry the samples by the Uni-pucks since June 2010. We can estimate how many samples had been mounted during a single beam time in the past three years (from April 2010 to December 2012), as well as the number of cassettes carried by the users. Almost all users inserted samples using four of fewer Uni-pucks, or one SSRL cassette (Fig. 3 ▶). A maximum of eight Uni-pucks were brought in during a single beam time (Fig. 3*a* ▶), and they could be inserted using the adapter cassettes inside the PAM liquid-nitrogen Dewar, because the PAM Dewar could hold 12 Uni-pucks simultaneously. On the other hand, a maximum of seven SSRL cassettes were brought in during another beam time (Fig. 3*b* ▶), but four or more SSRL cassettes could not be set inside the PAM Dewar simultaneously. Therefore, the experiments had to be interrupted to replace the cassettes.

## 3. Expansion of the AR-NE3A PAM capacity

From our estimate of the number of SSRL cassettes in a given beam time, we found that three or more SSRL cassettes were inserted just in AR-NE3A (Fig. 3*b* ▶). AR-NE3A is mainly used by pharmaceutical companies, and most of its samples are part of structural analysis investigations of the complexes of medicinal candidate compounds and target proteins. Therefore, we have decided to enlarge the capacity of the AR-NE3A PAM.

### 3.1. Larger liquid-nitrogen Dewar

**Figure 4:**
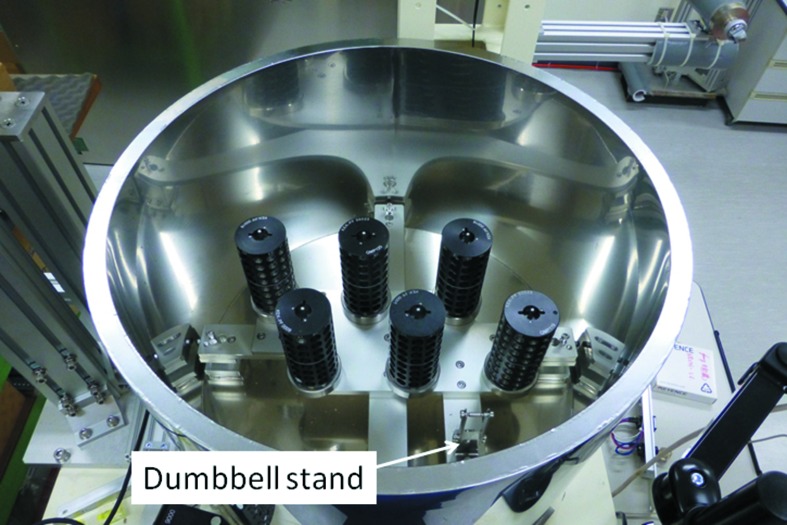
A larger liquid-nitrogen Dewar and cassette stand. There are six cassette locations within the developed Dewar. One dumbbell stand is attached to the cassette stand. One more dumbbell stand can be attached at a point-symmetrical position around the centre of the Dewar.

Since seven SSRL cassettes were used in only one beam time, the number of cassette locations we picked was six. A trial Dewar is shown in Fig. 4 ▶. Since easy manufacturing was desired, the trial Dewar was chosen to be cylindrical and made of stainless steel. This new Dewar has internal and external walls, with vacuum between the walls for heat insulation. The internal diameter of the trial Dewar is 550 mm, and the external diameter is 590 mm. The depth of the currently used commercial Dewar is 673 mm, but the lower half is unnecessary. Therefore, the depth of the trial Dewar is 360 mm and the overall height is 450 mm. Approximately 62 l of liquid nitrogen are necessary for keeping the samples at the cryogenic temperature (currently 57 l).

### 3.2. Cassette stand

The plate with six cassette locations is supported by four legs, attached by nuts welded to the bottom of the Dewar. Therefore, the screw heads attached to the side of the plate are unnecessary. The position of the three right-hand-side cassette locations and the dumbbell stand is almost the same as that of the existing system. This situation facilitates software development of the novel system. The left-hand three cassette locations are placed at point-symmetrical positions around the centre of the Dewar.

**Figure 5:**
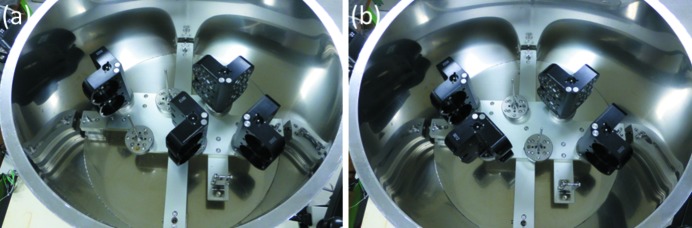
Examples of inserting the Uni-puck adapters. Five or six Uni-puck adapters cannot be placed simultaneously, because of interference.

Six SSRL cassettes can be inserted within the novel liquid-nitrogen Dewar (Fig. 4 ▶). However, only four Uni-puck adapters can be placed simultaneously. Figs. 5(*a*) and 5(*b*) ▶ show examples of inserted Uni-puck adapters. More adapters cannot be put on empty cassette locations in Fig. 5 ▶, because of interference.

### 3.3. Robot frame

**Figure 6:**
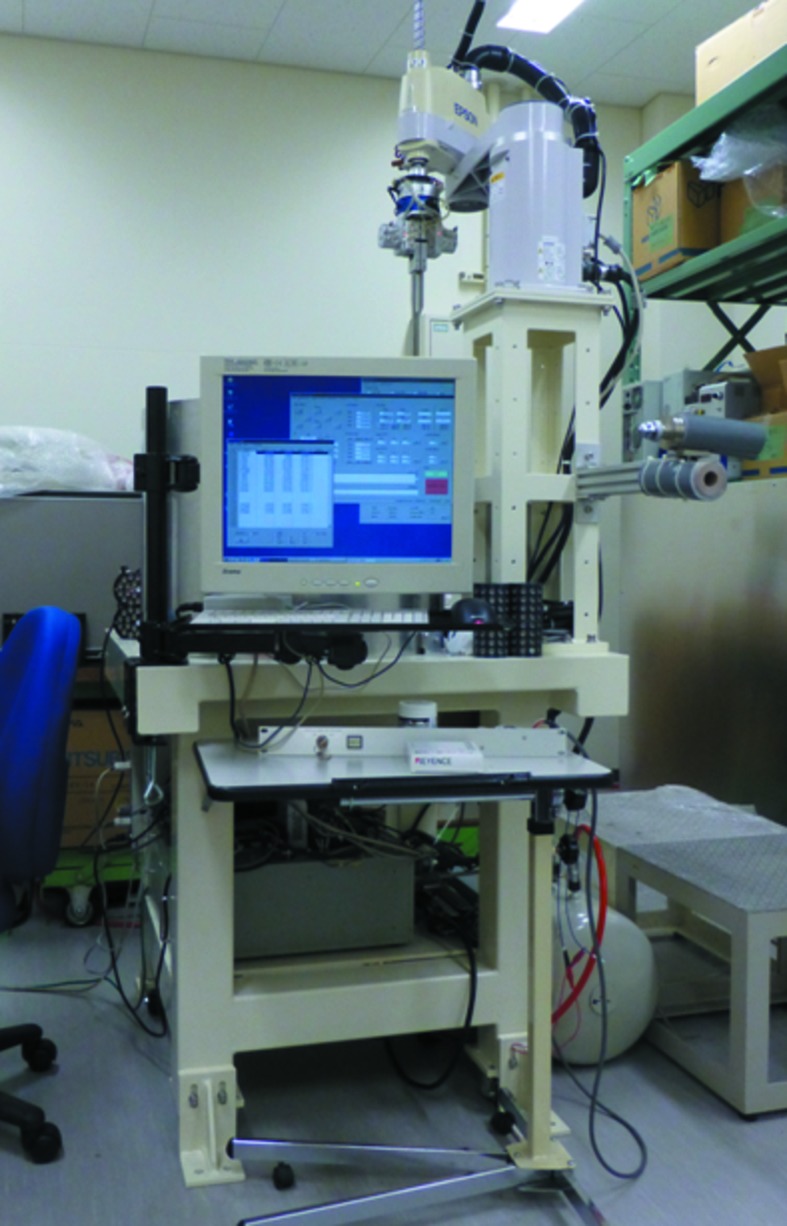
An industrial robot and a larger Dewar were implemented on the new frame.

Since the current frame of the AR-NE3A PAM was developed to use the commercial Dewar from Taylor Wharton, a robot frame was also designed from scratch to fit the larger Dewar. The new frame (Fig. 6 ▶) is larger, but the position of the frame legs is identical. We can easily install the new sample-exchange system with the new frame on AR-NE3A because we can use the same components to anchor the frame to the floor.

## 4. Calibration

For safe and stable operation, we calibrate the position and the orientation of the cassette stand components after the liquid nitrogen is replaced every two or three weeks. In addition, there is a need to recalibrate when there are malfunctions. In the calibration, the sample-exchange system automatically measures the position of the dumbbell stand, the parameters of the double tongs, the position and the orientation of each cassette, and the coordinates of the cryo-pin on the goniometer. We need to check whether the calibration is satisfactory using the new Dewar.

After filling the empty Dewar with liquid nitrogen and the cassette system becoming sufficiently cold, we calibrated the system. We then extracted the liquid nitrogen from the Dewar and dried it. We repeated this procedure four times and compared the calibration results for the existing and new systems. The calibration experiments for the existing system were carried out at BL-1A.

## 5. Results and discussions

**Figure 7:**
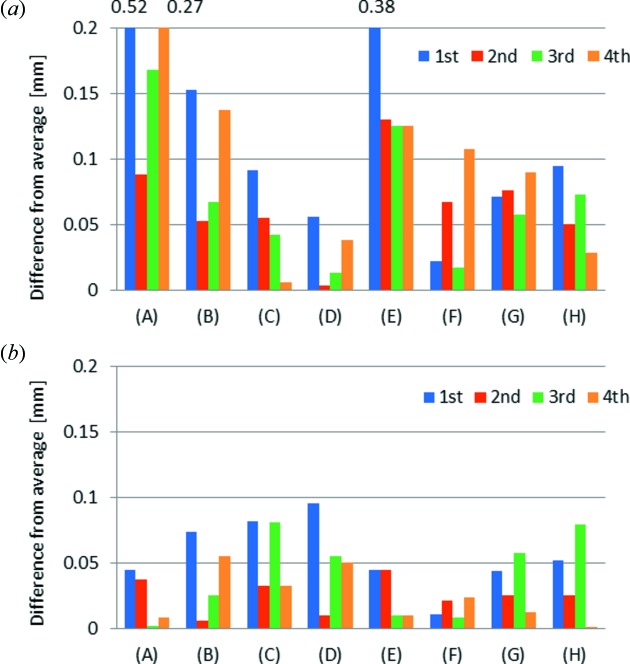
Comparison between the (*a*) existing and (*b*) new Dewars. (A)–(H) are calibration parameters: (A), (B) and (C) are the *x*-, *y*- and *z*-coordinates of the dumbbell stand, (D) and (E) are the double tong parameters A and B. (F), (G) and (H) are the *x*-, *y*- and *z*-coordinates of one of the cassettes.

Fig. 7 ▶ shows the results of the calibration experiments, comparing absolute values of the difference from the average of each parameter obtained by four calibration experiments. Since the parameters obtained by four calibration experiments at BL-1A differ greatly (Fig. 7*a* ▶), the calibration had to be carried out each time after drying the Dewar and adding liquid nitrogen. As described in §2.1, the cassette stand was not fixed firmly. The components of the cassette stand and the Dewar shrank after adding liquid nitrogen causing the cassette stand to move slightly, because not all components shrank equally. On the other hand, the change of the parameters for the new cassette stand and the Dewar was small (Fig. 7*b* ▶). From experience, an error below 0.1 mm does not cause a problem in the operation of the robot. Once the calibration of the new Dewar and cassette stand was carried out, we may not need to recalibrate, even if liquid nitrogen is added to an empty Dewar. This is a great advantage for maintaining a sample-exchange system, because the new Dewar and cassette stand have a lot of parameters which must be determined by calibration.

## 6. Conclusion

A larger liquid-nitrogen Dewar was manufactured, doubling the capacity of the sample-exchange system, allowing longer uninterrupted operation of fully automated experiments. Compared with the current system, the change in the parameters determined by calibration is very small, because the cassette stand can be fixed to the new Dewar permanently. This is not only useful for stable operation of the developed sample-exchange system but also facilitates maintenance of the whole system.

## References

[bb1] Chavas, L. M. G., Matsugaki, N., Yamada, Y., Hiraki, M., Igarashi, N., Suzuki, M. & Wakatsuki, S. (2012). *J. Synchrotron Rad.* **19**, 450–454. 10.1107/S0909049512009727 22514184

[bb2] Cipriani, F., Felisaz, F., Launer, L., Aksoy, J.-S., Caserotto, H., Cusack, S., Dallery, M., di-Chiaro, F., Guijarro, M., Huet, J., Larsen, S., Lentini, M., McCarthy, J., McSweeney, S., Ravelli, R., Renier, M., Taffut, C., Thompson, A., Leonard, G. A. & Walsh, M. A. (2006). *Acta Cryst.* D**62**, 1251–1259. 10.1107/S0907444906030587 17001102

[bb3] Cohen, A. E., Ellis, P. J., Miller, M. D., Deacon, A. M. & Phizackerley, R. P. (2002). *J. Appl. Cryst.* **35**, 720–726. 10.1107/s0021889802016709 PMC4041710 24899734

[bb4] Hiraki, M., Watanabe, S., pHonda, N., Yamada, Y., Matsugaki, N., Igarashi, N., Gaponov, Y. & Wakatsuki, S. (2008). *J. Synchrotron Rad.* **15**, 300–303. 10.1107/S0909049507064680 PMC2394784 18421164

[bb5] Hiraki, M., Watanabe, S., Yamada, Y., Matsugaki, N., Igarashi, N., Gaponov, Y. & Wakatsuki, S. (2007). *AIP Conf. Proc.* **879**, 1925–1928.

[bb6] Igarashi, N., Matsugaki, N., Yamada, Y., Hiraki, M., Koyama, A., Hirano, K., Miyoshi, T. & Wakatsuki, S. (2007). *AIP Conf. Proc.* **879**, 812–815.

[bb7] Jacquamet, L., Joly, J., Bertoni, A., Charrault, P., Pirocchi, M., Vernede, X., Bouis, F., Borel, F., Périn, J.-P., Denis, T., Rechatin, J.-L. & Ferrer, J.-L. (2009). *J. Synchrotron Rad.* **16**, 14–21. 10.1107/S0909049508031105 19096169

[bb8] Karain, W. I., Bourenkov, G. P., Blume, H. & Bartunik, H. D. (2002). *Acta Cryst.* D**58**, 1519–1522. 10.1107/s0907444902012751 12351852

[bb9] Ohana, J., Jacquamet, L., Joly, J., Bertoni, A., Taunier, P., Michel, L., Charrault, P., Pirocchi, M., Carpentier, P., Borel, F., Kahn, R. & Ferrer, J.-L. (2004). *J. Appl. Cryst.* **37**, 72–77.

[bb10] Snell, G., Cork, C., Nordmeyer, R., Cornell, E., Meigs, G., Yegian, D., Jaklevic, J., Jin, J., Stevens, R. C. & Earnest, T. (2004). *Structure*, **12**, 537–545. 10.1016/j.str.2004.03.011 15062077

[bb11] Ueno, G., Hirose, R., Ida, K., Kumasaka, T. & Yamamoto, M. (2004). *J. Appl. Cryst.* **37**, 867–873.

[bb12] Yamada, Y., Hiraki, M., Sasajima, K., Matsugaki, N., Igrashi, N., Amano, Y., Warizaya, M., Sakashita, H., Kikuchi, T., Mori, T., Toyoshima, A., Kishimoto, S. & Wakatsuki, S. (2010). *AIP Conf. Proc.* **1234**, 415–418.

